# Factors Affecting Persistent Postoperative Pain in Patients with Hip Fractures

**DOI:** 10.1155/2020/8814290

**Published:** 2020-11-04

**Authors:** Kyo Goto, Hideki Kataoka, Ayana Honda, Junichiro Yamashita, Kaoru Morita, Tatsuya Hirase, Junya Sakamoto, Minoru Okita

**Affiliations:** ^1^Department of Rehabilitation, Nagasaki Memorial Hospital, Nagasaki, Japan; ^2^Department of Physical Therapy Science, Nagasaki University Graduate School of Biomedical Sciences, Nagasaki, Japan; ^3^Department of Orthopedic Surgery, Nagasaki Memorial Hospital, Nagasaki, Japan; ^4^Institute of Biomedical Sciences, Nagasaki University, Nagasaki, Japan

## Abstract

Osteoporotic fractures are common among older people, and hip fractures (HF) can be devastating. Surgery is indicated for most cases of HF, and chronic persistent postoperative pain is likely to occur. This study investigated the multifaceted factors related to persistent pain occurring during the acute phase and subacute phase of recovery after HF surgery. We conducted a prospective 8-week study of older HF patients after surgery. We evaluated pain intensity, depression symptoms, the fear of falling, pain catastrophizing, cognition and attention, the ability to perform activities of daily living, and the physical performance at 2 weeks (acute phase) and at 4 weeks (subacute phase) after surgery. Patients were divided into the light group (Verbal Rating Scale (VRS) score ≤1) and severe group (VRS score ≥2) according to pain intensity at 8 weeks (recovery phase) after surgery. Factors affecting persistent postoperative pain during recovery were examined using logistic regression analysis. Seventy-two patients were analyzed: 50 in the light group and 22 in the severe group. In the severe group, pain with movement and Pain Catastrophizing Scale scores were higher than those of the light group at 2 weeks and at 4 weeks after surgery. The regression analysis showed that pain with movement at 2 weeks and at 4 weeks after surgery and pain catastrophizing at 4 weeks after surgery were related to persistent postoperative pain. HF patients may have persistent pain if they continue to experience pain and catastrophize their pain during the acute phase and subacute phase after surgery.

## 1. Introduction

Osteoporotic hip fractures (HF) are an unfortunate but common source of morbidity and mortality in elderly individuals. HF often occurs due to trauma caused by falling [1]. In 1999, HF occurred in 338,000 individuals who are 65 years and older, and this number is expected to exceed 500,000 by 2040 worldwide [2]. In many cases, after HF surgery, pain occurs in the area around the fracture and wound; however, it disappears due to bone fusion and healing of the wound. There are cases, however, in which the pain remains even after healing progresses [3]. It has been reported that some HF patients have chronic persistent pain at 6 months after surgery [4]. Chronic persistent postoperative pain is associated with disability [5]. Previous studies have suggested that postoperative pain in HF patients impairs physical function and the ability to perform activities of daily living (ADL) [6].

Cross-sectional research has shown that factors related to chronic persistent postoperative pain include severe pain, cognitive impairment, and psycho-emotional problems such as depression and catastrophizing [7, 8]. Additionally, it is necessary to deal with factors related to chronic persistent postoperative pain in the acute phase to prevent chronic pain. Longitudinal reports of predictive factors for chronic persistent postoperative pain have indicated that severe acute pain after surgery occurs with various diseases [9]. Furthermore, the International Association for the Study of Pain fact sheet states that prompt reduction of acute pain is important for the prevention of chronic pain in the elderly [10]. Moreover, it has been reported that anxiety and catastrophizing are related to the intensity of acute pain after open-heart surgery and total knee arthroplasty [11, 12].

The reasons underlying failure to recover from acute and subacute episodes of chronic persistent pain after HF surgery are not yet understood. Furthermore, a consensus has not been achieved regarding which factors are most highly associated with poor outcomes and persistent postoperative pain for HF patients. We investigated the multifaceted factors occurring in the acute phase and subacute phase after surgery that were related to persistent postoperative pain during recovery.

## 2. Materials and Methods

This study was conducted from April 2014 to October 2018. Patients older than 65 years who were admitted to the emergency unit at Nagasaki Memorial Hospital in Nagasaki, Japan, with a femoral neck fracture or femoral trochanteric fracture after a fall diagnosed by radiography were included. Exclusion criteria for this study comprised the transfer from another hospital, acute exacerbation, repeat fracture, discharge within 4 weeks after surgery, inability to walk within 4 weeks after surgery, and decline in cognitive function causing an inability to understand the questionnaire. The study protocol was approved by the Research Ethical Committee at the Graduate School of Biomedical Sciences at Nagasaki University (approval number: 17011249). All patients provided signed consent.

Data were collected prospectively. Cognition and attention, depression symptoms, the fear of falling, pain catastrophizing, pain intensity, the ability to perform ADL, and the physical performance at 2 weeks and at 4 weeks after surgery (acute phase and subacute phase, respectively) were measured by a physical therapist. To determine the physical performance, handgrip strength was measured at 2 weeks and at 4 weeks after surgery; other standing and gait assessments were measured only at 4 weeks after surgery. Radiological measurements were performed by a physical therapist who received specialized training from an orthopedist.

### 2.1. Rehabilitation Protocol

During the preoperative phase, therapists instructed their patients on strengthening and range-of-motion exercises (upper extremities and lower extremities without fracture). On the day of surgery, no rehabilitative procedures were performed.

After surgery, patients were mobilized out of bed and stand-up or ambulation training was initiated, depending on the surgical treatment (internal fixation or prosthetic replacement). First, with the therapist providing instructions, the patient received assistance getting out of bed and into a chair. Then, weight-bearing (as tolerated) training and stand-up or ambulation training with moderate assistance were started. Next, patients were trained to perform ambulation with a walker. Finally, progressive ambulation with a *T*-cane or independent gait as tolerated and strength and balance training were started. The physical therapist dedicated more than 60 minutes daily to each patient.

### 2.2. Epidemiological Background

We measured each patient's height and weight to calculate the body mass index. We also recorded the age, sex, length of hospital stay, fracture type (femoral neck fracture or trochanteric fracture), and type of surgery (bipolar hip arthroplasty (BHA) or compression hip screw (CHS) or *γ* nail or pinning) of each patient. Additionally, we investigated the ability of each patient to perform basic ADL and instrumental ADL (IADL) before admission. The performance of basic ADL was determined using the Barthel index (BI) [13]. The BI was developed in 1955 as a simple index of independence and is useful for scoring disability [13, 14]. IADL were examined using the Tokyo Metropolitan Institute of Gerontology (TMIG) Index of Competence [15].

### 2.3. Pain Intensity

Pain intensity, which was defined as the strongest pain level of the day, was measured using a 5-point Verbal Rating Scale (VRS) (0, no pain; 1, light pain; 2, moderate pain; 3, severe pain; 4, intolerable pain). Pain at rest and pain during standing and walking were measured [16].

### 2.4. Depression Symptoms, Fear of Falling, and Pain Catastrophizing

The Pain Catastrophizing Scale (PCS) reported by Sullivan et al. includes 13 items and comprises three categories: rumination (five items), helplessness (five items), and magnification (three items). Each item is assessed using a 5-point scale ranging from 0 points (not at all) to 4 points (all the time), with a total score ranging from 0 to 52 points; higher scores indicated greater catastrophizing [17]. To assess depression symptoms, the Japanese short version of the 15-item Geriatric Depression Scale (GDS) was used [18, 19]. The Fall Efficacy Scale (FES) questionnaire was used to express the degree of concern about the possibility of falling during the execution of 10 ADL. The FES uses a four-level Likert Scale, and each level corresponds to a score ranging from 1 (not at all worried) to 4 (very worried) [20]. The individual scores are added together to calculate a total score ranging from 10 to 40.

### 2.5. Cognition and Attention

General cognitive function was measured using the Japanese version of the Mini-Mental State Examination (MMSE) [21, 22]. The trail making test part A (TMT-A) was used to assess visual search, attention, and motor speed tasks [23, 24].

### 2.6. Activities of Daily Living

We used the functional independence measure (FIM) to determine the generic ability to perform ADL. This performance-based disability tool assesses the level of disability when performing basic ADL [25–28].

### 2.7. Physical Performance

Handgrip strength (kg) was measured using a digital hand dynamometer (Smedley's Dynamometer; TTM, Tokyo, Japan) in the standing position; a maximum of two measurements of the dominant hand was recorded [29].

During the sit-to-stand (5STS) test, patients were asked to stand up and sit down as quickly as possible five times with their arms crossed in front of their chest. The floor-to-seat height was 45 cm [30]. The timed up and go (TUG) test was performed by timing the ability of the patients to stand up from a chair, walk 3 m, turn around, walk back to the chair, and sit down [31].

The six-minute walking test (6-MWT) was used to assess if the functional exercise capacity correlated with physical fitness. This test measures the distance (in meters) that a patient can quickly walk on a flat, hard surface during a period of 6 minutes [32].

### 2.8. Statistical Analysis

During this study, persistent postoperative pain was defined as pain that persisted for 2 months (8 weeks) after surgery (the recovery phase). Pain with movement scores at 8 weeks after surgery were analyzed as no pain or light pain (VRS score = 0-1; light group) or moderate to severe pain (VRS score = 2-3; severe group); and no patients reported intolerable pain (VRS score = 4). Based on previous research [16], all data were examined statistically for normality of distribution (Kolmogorov–Smirnov).

An unpaired *t*-test, Mann–Whitney *U* test, and chi-square test were used to compare the basic survey items of the light group and severe group at each evaluation time point. After comparisons were performed, a logistic regression analysis was performed to examine factors affecting persistent postoperative pain associated with HF. All significance levels were set less than 5%.

## 3. Results

**Figure 1:**
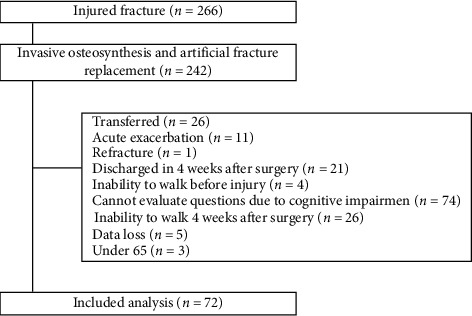
Flow chart outlining the number of participants in each arm of the study.

From January 2014 to October 2018, 266 patients were admitted to our hospital with a fracture of the proximal femur and 242 patients underwent open osteosynthesis and BHA. The following were excluded: patients transferred from other hospitals, patients with acute exacerbation, patients with repeat fracture, patients discharged within 4 weeks after surgery, patients unable to walk before injury, patients who could not comprehend questions due to cognitive impairment, patients unable to walk 4 weeks after surgery, patients younger than 65 years, and patients without sufficient data. As a result, 72 patients were analyzed (Figure 1).

Regarding pain with movement at 8 weeks after surgery, of the 72 total patients, 30 (41.7%) had a VRS score of 0, 20 (27.8%) had a VRS score of 1, 15 (20.8%) had a VRS score of 2, and 7 (9.7%) had a VRS score of 3. Therefore, 50 (69.4%) patients were in the light group and 22 (30.6%) were in the severe group.

### 3.1. Patient Characteristics

There were no significant differences between the light group and severe group regarding age, sex, body mass index, length of hospital stay, fracture type, the prehospital ability to perform ADL, and the prehospital ability to perform IADL (Table 1).

### 3.2. Comparison of the Light and Severe Groups at 2 Weeks and at 4 Weeks after Surgery

At 2 weeks after surgery, the mean VRS scores for pain at rest were 0.29 ± 0.5 in the light group and 0.72 ± 1.2 in the severe group; the severe group had significantly higher values than the light group. Additionally, the mean VRS scores for pain with movement were 1.25 ± 1.1 in the light group and 2.14 ± 1.0 in the severe group; the severe group had significantly higher values than the light group. The GDS and FES scores were not significantly different between the light group and severe group. The mean total PCS scores were 20.5 ± 11.5 points in the light group 2 weeks after surgery and 26.4 ± 11.0 points in the severe group. The severe group had significantly higher scores than the light group.

The MMSE and TMT-A scores were not significantly different between the light group and severe group. The mean FIM score was not significantly different between the light group and severe group. There was no significant difference in grip strength between the light group and severe group (Table 2).

At 4 weeks after surgery, there was no significant difference in pain with movement in the light group and severe group. However, the mean VRS scores for pain with movement were 0.94 ± 1.94 in the light group and 2.13 ± 0.8 in the severe group. The severe group had significantly higher scores than the light group. The GDS and FES scores were not significantly different between the light group and severe group. The mean total PCS scores were 14.9 ± 10.3 points in the light group and 27.3 ± 10.1 points in the severe group. The severe group had significantly higher scores than the light group. The MMSE and TMT-A results were not significantly different between the light group and severe group. The mean FIM score was not significantly different between the light group and severe group. There was no significant difference in the results of grip strength, 5TST, TUG, and 6-MWT between the light group and severe group (Table 3).

### 3.3. Factors at 2 Weeks and at 4 Weeks after Surgery Related to Persistent Postoperative Pain

To investigate the factors related to persistent pain at 8 weeks after surgery, a logistic regression analysis was performed using pain with movement at 8 weeks after surgery as a dependent variable. Factors that showed significant differences between the two groups (2 weeks after surgery: age, sex, pain at rest, pain with movement, and total PCS score; 4 weeks after surgery: age, sex, pain with movement, and total PCS score) were used as independent variables. The factor affecting pain persistence at 8 weeks after surgery and at 2 weeks after surgery was pain with movement (odds ratio (OR), 1.91; 95% confidence interval (CI) 1.06-3.47). However, factors affecting pain persistence at 8 weeks after surgery and at 4 weeks after surgery were pain with movement (OR, 3.07; 95% CI, 1.33-7.06) and total PCS score (OR, 1.09; 95% CI, 1.02-1.17) (Table 4).

## 4. Discussion

We examined the multifaceted characteristics and pain-related factors in HF patients during the acute and subacute phases (2 weeks and 4 weeks, respectively) after surgery and determined the factors related to persistent postoperative pain during the recovery phase (8 weeks after surgery). A comparison of the light group and severe group suggested that severe pain and catastrophizing at 4 weeks after surgery were characteristics of patients with persistent postoperative pain. The regression analysis indicated that factors related to persistent postoperative pain were pain with movement at 2 weeks after surgery and pain with movement and PCS score at 4 weeks after surgery.

In this study, 22 of the 72 patients (30.6%) had severe pain at 8 weeks after surgery. In a previous study, 49% of the patients had persistent pain at 3 months after total knee arthroplasty [33]. Furthermore, 28% of patients had pain 3 months after total hip arthroplasty [34]. Therefore, the rate of persistent pain 8 weeks after HF surgery was similar to that reported by previous studies of lower limb surgery.

Pain with movement at 2 weeks (acute phase) and at 4 weeks (subacute phase) after HF surgery was related to persistent postoperative pain at 8 weeks. It has been reported that severe pain persisting 3 months after total knee arthroplasty cannot be controlled [35]. Additionally, it has been reported that patients who undergo surgery for lower limb fractures or thoracotomy (13%) can have chronic severe pain at 6 months if the pain cannot be reduced within 5 days after surgery [36]. Therefore, if severe pain persists during the acute phase after HF, then the pain is likely to continue during the recovery phase.

The PCS score and pain with movement at 4 weeks after surgery were factors related to pain at 8 weeks after surgery. Patients who underwent total knee arthroplasty [37] and lower extremity surgery [38] and catastrophized their pain at 4 weeks postoperatively had pain at 1 year after surgery. Therefore, persistent postoperative pain in HF patients during the recovery phase is related not only to pain intensity but also to catastrophizing pain during the acute phase. Pain tends to persist if it is catastrophized after surgery because high levels of catastrophizing may lead individuals to respond selectively and intensely to pain-related stimuli [39]. Furthermore, catastrophizing impacts the experience of pain and promotes sensitization or interference with pain inhibition in the descending pain inhibitory pathway of the central nervous system [40].

One of the limitations of this study was that the grouping based on the VRS score was not exact. We investigated factors affecting pain only during the acute and subacute phases; therefore, factors affecting pain during the chronic phase (3 to 6 months postoperatively) must be investigated. The types of surgical procedures performed for HF vary and are not uniform. Finally, the sample size was small and power analysis was not performed; therefore, the analysis was not sufficient.

## 5. Conclusions

For HF patients, pain with movement at 2 weeks and at 4 weeks after surgery and the PCS score at 4 weeks after surgery were related to persistent postoperative pain at 8 weeks after surgery (recovery phase). Therefore, if patients experience severe pain during the acute phase after HF surgery, and if severe pain and catastrophizing occur during the subacute phase, then persistent postoperative pain is likely to occur during the recovery phase. Interventions including psychological support are necessary during the acute phase to prevent persistent postoperative pain.

## Figures and Tables

**Table 1:** Patient characteristics.

	Light group (*n* = 50)	Severe group (*n* = 22)	*p* value
Age (years)	82.8 ± 6.5	82.9 ± 7.2	0.98
*Sex*			0.88
Male	5	2	
Female	44	20	
BMI	20.4 ± 3.2	21.5 ± 3.8	0.21
Length of hospital stay (days)	88.4 ± 26.6	90.9 ± 26.6	0.71
*Fracture type*			0.86
Femoral neck fracture (*n*)	30	13	
Trochanteric fracture (*n*)	19	9	
*Type of surgery*			0.37
BHA (*n*)	21	7	
CHS (*n*)	10	9	
*γ* nail (*n*)	12	4	
Pinning (*n*)	6	2	
Barthel Index before admission (point)	95.0 ± 7.6	90.5 ± 13.9	0.08
TMIG index before admission (point)	6.9 ± 4.2	6.6 ± 4.3	0.75

Values are expressed as mean ± standard deviation (SD). BHA, bipolar hip arthroplasty; BMI, body mass index; CHS, compression hip screw; TMIG, Tokyo Metropolitan Institute of Gerontology.

**Table 2:** Comparison between the groups at 2 weeks after surgery.

2 weeks after surgery	Light group	Severe group	*p* value
MMSE	23.3 ± 6.2	22.1 ± 6.1	0.46
TMT-A	166.1 ± 138.5	125.5 ± 66.1	0.25
Rest pain	0.29 ± 0.5	0.72 ± 1.2	0.04
Move pain	1.25 ± 1.1	2.14 ± 1.0	0.001
Total PCS	20.5 ± 11.5	26.4 ± 11.0	0.04
GDS	7.1 ± 4.0	7.8 ± 3.8	0.53
FES	24.0 ± 6.5	22.2 ± 7.8	0.34
Handgrip strength	14.9 ± 6.5	13.7 ± 4.5	0.42
FIM	77.8 ± 19.4	74.6 ± 15.9	0.50

Values are expressed as mean ± standard deviation (SD). FES, Fall Efficacy Scale; FIM, functional independence measure; GDS, Geriatric Depression Scale; MMSE, Mini-Mental State Examination; PCS, Pain Catastrophizing Scale; TMT-A, trail making test part A; VRS, Verbal Rating Scale. Signiﬁcant group difference (*p* < 0.05).

**Table 3:** Comparison of groups at 4 weeks after surgery.

4 weeks after surgery	Light group	Severe group	*p* value
MMSE	24.6 ± 5.2	22.7 ± 4.7	0.16
TMT-A	143.0 ± 69.7	150.5 ± 84.7	0.74
Pain at rest	0.22 ± 0.68	0.23 ± 0.61	0.98
Pain with movement	0.94 ± 1.94	2.13 ± 0.8	<0.01
Total PCS	14.9 ± 10.3	27.3 ± 10.1	<0.01
GDS	7.1 ± 4.0	7.8 ± 4.3	0.08
FES	26.7 ± 5.6	23.6 ± 6.8	0.05
Handgrip strength	15.7 ± 6.1	14.25 ± 5.1	0.34
5STS	20.5 ± 16.2	21.4 ± 8.5	0.8
TUGT	33.5 ± 27.6	40.6 ± 24.0	0.3
6-MWT	154.2 ± 103.7	113.8 ± 89.0	0.12
FIM	93.3 ± 18.9	90.6 ± 14.86	0.56

Values are expressed as mean ± standard deviation (SD). 5STS, sit-to-stand test; 6-MWT, six-minute walking test; FES, Fall Efficacy Scale; FIM, functional independence measure; GDS, Geriatric Depression Scale; MMSE, Mini-Mental State Examination; PCS, Pain Catastrophizing Scale; TMT-A, trail making test part A; TUG, timed up and go test; VRS, Verbal Rating Scale. Signiﬁcant group difference (*p* < 0.05).

**Table 4:** Factors affecting persistent postoperative pain at 2 weeks and 4 weeks after HF surgery.

Partial regression				95% CI	
Variable	Coefficient	*p* value	OR	Lower limit	Upper limit
*2 weeks after surgery*					
Age	0.98	0.67	0.98	0.89	1.07
Sex	0.2	0.7	1.5	0.2	11.08
Pain at rest	0.26	0.47	1.3	0.64	2.64
Pain with movement	0.65	0.03	1.91	1.06	3.47
Total PCS	0.03	0.31	1.02	0.98	1.08
*4 weeks after surgery*					
Age	−0.002	0.97	1.0	0.89	1.12
Sex	0.11	0.87	1.24	0.08	18.43
Pain with movement	1.12	0.008	3.07	1.33	7.06
Total PCS	0.09	0.012	1.09	1.02	1.17

CI, confidence interval; OR, odds ratio; PCS, Pain Catastrophizing Scale; VRS, Verbal Rating Scale.

## Data Availability

The datasets used to support the findings of this study are available from the corresponding author upon request.
